# Shared Pathophysiology of Inflammatory Bowel Disease and Psoriasis: Unraveling the Connection

**DOI:** 10.7759/cureus.70148

**Published:** 2024-09-25

**Authors:** Shadi Tabbarah, Hakam Sulaiman, Frank Ansah Owusu, Megha Rajeev Joshi, Nitheesha Reddy Marepalli, Nohelia Pino, Samra Saleem Azam, Aaliya Ali Ahmed, José Abraham Suárez Álvarez

**Affiliations:** 1 Department of Medicine, Lebanese American University School of Medicine, Beirut, LBN; 2 Department of Medicine, American University of the Caribbean School of Medicine, Cupecoy, SXM; 3 Department of Medicine, Stavropol State Medical University, Stavropol, RUS; 4 Department of Medicine, West Pine Medical, St. Louis, USA; 5 Department of Medicine, Smt. Nathiba Hargovandas Lakhmichand (NHL) Municipal Medical College, Ahmedabad, IND; 6 Department of Medicine, Dr. Patnam Mahender Reddy (PMR) Institute of Medical Sciences, Hyderabad, IND; 7 Department of Medicine, University of Manizales, Manizales, COL; 8 Department of Medicine, Dow Medical College, Karachi, PAK; 9 Department of Internal Medicine, Aga Khan Hospital Mombasa, Mombasa, KEN; 10 Department of General Surgery, Tijuana General Hospital, Tijuana, MEX

**Keywords:** autoimmune diseases, genetic susceptibility, il-23/th17 pathway, immunopathogenesis, inflammatory bowel disease (ibd), psoriasis

## Abstract

Inflammatory bowel disease (IBD) and psoriasis are both chronic autoimmune diseases with a unique set of characteristics. Interestingly, both conditions share considerable overlap in their pathophysiological mechanisms and immune dysregulation. Epidemiological studies validate the relationship by showing a greater prevalence of co-occurrence of the two disorders. At the genetic level, there is a confirmation of a link between shared susceptibility loci and DNA polymorphism, particularly interleukin-23 receptor (IL23R), interleukin-12 subunit beta (IL12B), tumor necrosis factor (ligand) superfamily member 15 (TNFSF15), and signal transducer and activator of transcription 3 (STAT3). In addition, epigenetic factors have a role in genetic predisposition in the development and progression through processes such as DNA methylation and histone modification adding another layer of genetic susceptibility. The relationship between psoriasis and IBD is emphasized by a comparable immunopathogenesis, which involves delicate relationships between the innate and adaptive immune responses. The primary interest is on the T-helper 17 (Th17) cell pathway and the cytokines interleukin-17 (IL-17), interleukin-23 (IL-23), and tumor necrosis factor-alpha (TNF-α). Consequently, both disorders exhibit chronic inflammation and tissue restructuring, resulting from similar cellular and molecular processes. The presence of overlapping pathophysiology highlights the significance of implementing integrated management strategies and employing multidisciplinary techniques for both diagnosis and therapy. Hence, understanding the mutual processes might facilitate the advancement of precise biologic treatments that aim at these commonly shared inflammatory pathways.

## Introduction and background

Inflammatory bowel disease (IBD) is a chronic condition characterized by inflammation primarily in the colon and small intestine, following a relapsing-remitting course. IBD encompasses two main conditions, Crohn's disease (CD) and ulcerative colitis (UC), which present with symptoms like weight loss, diarrhea, abdominal pain, and rectal bleeding. Although CD and UC share some immunological processes, their pathogenesis differs [1]. CD affects the entire digestive tract, from the mouth to the anus, involving all layers of the intestinal wall, while UC is confined to the mucosal layer of the colon [2]. The two conditions also target different regions of the digestive system; CD often involves the ileum and parts of the large intestine, whereas UC is restricted to the colon and rectum. While UC spares the small intestine, CD frequently causes inflammation in this area [3]. Given that both CD and UC are significant subtypes of IBD, CD is particularly linked to severe skin complications [2].

Psoriasis presents with several key signs and symptoms, including erythematous scaly patches or plaques, typically seen on extensor surfaces such as the elbows and knees, but it may also affect areas like the scalp, trunk, and nails. Patients often experience itching, burning, or pain in these areas, with plaques that vary in size, thickness, and distribution​ [4,5]. Nail involvement is common, presenting with features such as pitting, onycholysis, and dystrophy. Psoriatic arthritis can develop in a subset of patients and is often associated with joint stiffness, pain, and swelling​ [5]. Psoriasis is not only a dermatological condition but also linked to several systemic comorbidities such as cardiovascular disease, diabetes, obesity, and mental health disorders including depression and anxiety​ [4,5]. These associations likely result from shared genetic predispositions, systemic inflammation, or overlapping pathogenic pathways rather than the duration of the disease alone​ [5].

The treatment of psoriasis includes both topical and systemic therapies, depending on disease severity. For mild cases, topical treatments remain the cornerstone and include corticosteroids, vitamin D analogues, and calcineurin inhibitors [4,5]. For moderate to severe cases, conventional systemic treatments like methotrexate, cyclosporine, and acitretin are employed. However, biologic therapies targeting key inflammatory cytokines such as tumor necrosis factor-alpha (TNF-α), interleukin-17 (IL-17), and interleukin-23 (IL-23) have significantly advanced the management of moderate to severe psoriasis​ [4,5]. These biologics are effective in reducing the extent of the disease and are often preferred for patients with extensive plaque psoriasis or associated psoriatic arthritis.

Psoriasis and IBD are closely linked due to their shared inflammatory nature. The pathogenesis of both psoriasis and IBD involves shared genetic and immunological mechanisms. Both conditions are characterized by chronic inflammation, primarily driven by the dysregulation of immune responses. Psoriasis is largely associated with the activation of T-helper 17 (Th17) cells, which produce pro-inflammatory cytokines such as IL-17 and IL-22. These cytokines promote skin inflammation and keratinocyte proliferation, leading to the hallmark plaques of psoriasis [6-8]. Similarly, IBD, particularly CD, involves Th17-mediated immune responses that lead to inflammation of the gastrointestinal tract​ [6,8]. Genetic studies have identified several shared susceptibility loci between psoriasis and IBD, including 6p22, 16q, and 5q33, which are linked to immune system regulation​ [6,8]. The microbiome also plays a significant role in both diseases, with a reduction in beneficial bacteria observed in affected patients [6].

These conditions are often considered homogenous due to their overlapping genetic predispositions, immune mechanisms, and therapeutic approaches. Both diseases involve cytokines such as TNF-α and IL-23, making anti-TNF and IL-23 inhibitors effective in managing moderate to severe cases of psoriasis and IBD​ [6]. The use of common biological therapies underscores the shared pathogenic pathways and validates the clinical and molecular similarities between these diseases. However, it is important to note that while they share common elements, the expression and progression of psoriasis and IBD can vary significantly depending on environmental factors and the specific genetic background of the individual [6].

This scoping review aims to assess the gathered information concerning the relationship between IBD and psoriasis as well as the immunological mechanisms associated with the two diseases. Furthermore, this study summarizes the available information on the clinical association, genetic and epigenetic linkages, pathophysiological mechanisms, as well as overlapping treatments of both diseases and highlights the current research and emerging directions of the two diseases.

## Review

Epidemiology and clinical association

Psoriasis and IBD, that is, UC and CD, have a well-documented epidemiological and clinical association. In a recent meta-analysis including 93 studies, it was found that the prevalence of psoriasis among patients with CD was 3.6% (95% confidence interval (CI): 3.1-4.6%) while among patients with UC was 2.8% (95% CI: 2-3.8%) [9]. Conversely, the prevalence of CD and UC in patients with psoriasis was 0.7% (95% CI: 0.2-1.3%) and 0.5% (95% CI: 0.3-0.8%), respectively [9]. This highlights a significant linkage between psoriasis and IBD. 

The repeated concurring of psoriasis with gastrointestinal diseases such as UC and CD has been highlighted by multiple studies [10-12], suggesting a shared pathophysiological basis and genetic markers. For instance, Urun Unal et al. conducted a study involving 111 psoriasis patients aged 18-80 years, which established that irritable bowel syndrome (IBS) was notably more common among psoriasis groups in comparison with a control group of healthy individuals. This correlation is attributed to shared genetic markers and pathophysiological factors [10].

The General Hospital Medical Center Leeuwarden (MCL) in the Netherlands conducted a retrospective cohort study on the prevalence of psoriasis and its various phenotypes, considering factors like sex, age of onset, body mass index, medications, and comorbidities. This study found a higher prevalence of psoriasis-IBD cases among females (60%) and noted that younger age at onset in psoriasis-CD phenotypes often correlates with milder forms of psoriasis [11]. Additionally, a meta-analysis was done for four case-control studies that found psoriasis patients are 1.71 times more likely to have UC while 2.53 times more likely to have CD than controls [12].

Sex-specific differences in disease manifestation are also observed [10,13]. A study found that psoriasis patients have a higher risk of CD and UC, with younger women more prone to CD and men more likely to develop UC. Psoriatic arthritis increases the risk of both conditions, while biologics seem to mitigate this risk by managing IBD symptoms​ [13].

In the generalized US population, CD prevails at seven per 100,000, with siblings of affected individuals facing a 3-5% risk, underscoring a familial predisposition [8,13]. Immunological diseases often cluster within families, increasing the probability of developing similar pathologies among family members [8].

Genetic links between IBD and psoriasis 

The two inflammatory ailments of a chronic nature, psoriasis and IBD, have been linked by genetic susceptibility loci. Psoriasis occurs more frequently in CD patients than would be expected if the two conditions were entirely independent [14], hence growing research on shared genetic links among the diseases. The genetic susceptibility loci for psoriasis and IBD are located on chromosome 6p21; these loci correspond to Psoriasis gene 1 (PSORS1) in psoriasis and Inflammatory Bowel Disease 3 (IBD3) [12]. This chromosomal locus which encompasses the major histocompatibility complex (MHC)-related genes has been extensively studied. Additionally, significant genetic loci unrelated to the MHC have been identified, including Interleukin-23 receptor (IL23R), interleukin-12B (IL12B), tumor necrosis factor superfamily member 15 (TNFSF15), and signal transducer and activator of transcription 3 (STAT3). Due to its key role in the synthesis of pro-inflammatory cytokines such as IL-22 and IL-17, IL23R is regarded as the "master regulator" of the immune-inflammatory response [15,16]. Another locus, IL12B, encodes a subunit of IL-12, which aids in T-helper 1 (Th1) cell differentiation, essential for immune responses. TNFSF15 regulates immune responses and is linked to both IBD and psoriasis, while STAT3, which encodes a transcription factor activated by cytokines and growth factors, influences cell proliferation and apoptosis [17]. These shared genetic loci offer a unified approach to the treatment of psoriasis and IBD, with the skin and bowel serving as both barriers and connections between the body's internal and external environments [8].

Genome-wide association studies (GWAS) have identified numerous shared genetic regions between IBD and psoriasis, particularly highlighting key loci such as IL23R, IL12B, REL, and tyrosine kinase 2 (TYK2). These loci are involved in immune regulation and inflammatory pathways, which are central to the pathogenesis of both diseases​ [7]. These discoveries have significantly advanced the development of targeted treatments, such as biologic therapies that block specific pro-inflammatory cytokines like IL-17 and IL-23, offering more effective and personalized treatment options for patients with psoriasis and IBD​ [7].

Studies involving twins and familial aggregation offer important new insights into the genetic and environmental variables influencing the onset of conditions such as IBD and psoriasis. Psoriasis and CD have a significant hereditary tendency, as indicated by the high concordance between the two disorders in twins [6]. In IBD, monozygotic twins show higher concordance rates than dizygotic twins, indicating significant genetic influence, though environmental factors also play a role [6,18,19]. Similarly, psoriasis exhibits around 70% concordance in monozygotic twins, with lower rates in dizygotic twins, and high prevalence among first-degree relatives, with recurrence risks ranging from 4 to 18.5 [18]. However, studies have indicated that first-degree relatives of IBD patients are at a significantly elevated risk of developing the illness than the general population [19]. Compared to UC, the risk of CD has been greater [19].

Immunopathogenesis 

There are clear parallels in the immunopathogenesis of IBD and psoriasis, particularly in the dysregulated immune responses, dendritic cell activation, and T-cell-mediated cytokine proliferation observed in both conditions [7,20,21]. The chronic inflammatory nature of both diseases is driven by a combination of genetic, environmental, and immune factors [7,20-22]. Persistent inflammation and immune dysregulation are critical drivers of the pathological mechanisms in both conditions, primarily involving T cells and elevated levels of pro-inflammatory cytokines such as IL-23, IL-17, and TNF-α [7,8].

TNF-α, a key mediator of inflammation, promotes the infiltration of immune cells into tissues by inducing the expression of adhesion molecules on endothelial cells. This results in a compromised barrier function, leading to further inflammation and tissue damage, as seen in CD and psoriasis [22,23]. The efficacy of TNF-α inhibitors like adalimumab and infliximab in treating both conditions highlights the critical role of this cytokine in their pathogenesis [24]. These biologic agents target and neutralize TNF-α, thereby reducing inflammation and improving clinical outcomes [24-26].

In addition to TNF-α, dendritic cells play a pivotal role in regulating immune responses in both psoriasis and IBD. Through pattern recognition receptors (PRRs), dendritic cells detect pathogens and modulate the production of IL-23, which in turn stimulates Th17 cells to produce IL-17, driving inflammation [7,25]. This IL-23/Th17 axis is crucial in both diseases, as it leads to sustained inflammation, keratinocyte hyperproliferation in psoriasis, and tissue damage in IBD [27-29]. Genetic polymorphisms in the IL23R gene further underline the shared immunopathogenic mechanisms between these diseases [30].

The comorbidity of these conditions may be associated with the loss of anti-pro-inflammatory signals. Additional findings show that psoriasis patients exhibit a deficiency in IL-10, a crucial component of the regulatory immune process [7]. IL-10 exhibits key anti-inflammatory actions via the downregulation of macrophage and dendritic cell response, indirectly limiting T-cell activation [1,31]. This is further supported by the usage of anti-TNF medication that disrupts the response of the immune system by the reduction in the making of IL-17, TNF-α, and IFN-γ, after stimulating with anti-CD28 and anti-CD3 in a laboratory setting [24]. TNF inhibitors have unique modes of action, whereby anti-TNF agents exert a rapid effect on Th17 populations and demonstrate a higher inhibition of Th1 populations, which is strongly associated with clinical improvement [24]. Together, it is evident that a dysregulation within homeostasis levels of pro-inflammatory cytokines and anti-inflammatory signals can be attributed to both disease pathogenesis [7].

Effects of conventional and biologic treatments

The treatment of both psoriasis and IBD relies on targeting shared inflammatory pathways, especially those involving TNF-α, IL-23, and IL-17. Conventional systemic anti-inflammatory and immunosuppressive treatments have been widely used in both conditions. Drugs such as cyclosporine, methotrexate, and azathioprine have been effective in managing symptoms, although their long-term use is associated with significant side effects, including increased infection risks, hepatotoxicity, and bone marrow suppression [32,33].

Biologic Agents

Biologics have revolutionized the treatment of psoriasis and IBD by offering more targeted therapies. TNF-α inhibitors (e.g., infliximab and adalimumab) are FDA-approved for both psoriasis and IBD and have shown great efficacy in reducing inflammation and achieving remission​ [32]. However, some patients experience paradoxical reactions, where psoriasis may worsen or new cases of IBD may emerge during treatment​ [32]. TNF-α inhibitors are generally considered safe for both diseases, but close monitoring is required to mitigate the risk of paradoxical reactions.

IL-12/IL-23 Inhibitor

Ustekinumab, which targets the IL-12/IL-23 pathway, is effective in treating both psoriasis and CD, although its effects on UC are still being explored. Ustekinumab is particularly beneficial for patients with both conditions, as it can reduce inflammation in both the skin and gut​ [32]. Clinical trials and real-world studies have shown sustained remission in CD and psoriasis patients receiving ustekinumab, with a favorable safety profile​ [32,33].

IL-17 Inhibitors

While IL-17 inhibitors, such as secukinumab and ixekizumab, have proven effective in treating psoriasis, their use in IBD is more controversial. These agents have been associated with exacerbating IBD symptoms or triggering new cases, particularly in patients with a history of CD [33]. This makes IL-17 inhibitors less favorable for patients with coexisting psoriasis and IBD, despite their efficacy in managing psoriasis symptoms [32].

IL-23 Inhibitors

Guselkumab and risankizumab, which target the IL-23 pathway, are newer biologics that have shown promise in treating psoriasis. Their role in IBD management is still under investigation, with early studies suggesting potential benefits for patients with CD​ [32].

Biologic treatments offer tailored approaches for managing psoriasis and IBD, but their selection must consider the risk of paradoxical reactions and disease exacerbation, especially with IL-17 inhibitors. TNF-α and IL-12/IL-23 inhibitors remain the most reliable options for treating both conditions, while newer agents like IL-23 inhibitors hold promise but require further validation through clinical trials​ [32,33].

Environmental and epigenetic factors

Environmental factors play a significant role in the development and exacerbation of psoriasis and IBD. Increased incidence rates in industrialized areas are likely due to lifestyle changes, with smoking, diet, and pollution identified as key environmental triggers. Smoking has contrasting effects on CD and UC; smokers are at higher risk for CD, while ex-smokers have a greater risk for UC [34]. Moreover, individuals exposed to higher levels of environmental pollutants, such as NO2 and SO2, show a higher risk of developing these conditions [35].

Diet is another important environmental factor, with high-fat, carbohydrate-rich, and animal protein-heavy diets increasing the risk of IBD, while diets high in fiber, fruits, and vegetables offer protection [36]. Nonsteroidal anti-inflammatory drugs (NSAIDs), including aspirin, are also associated with an increased risk of CD due to their inhibition of protective prostaglandins and promotion of ischemic environments that sustain chronic inflammation [37].

Epigenetic factors, such as DNA methylation, histone modification, and non-coding RNA expression, have been implicated in both psoriasis and IBD [22]. Epigenetic modifications in genes related to immune regulation, such as IL23R and IL12B, contribute to the altered immune responses seen in both diseases [22,30]. These findings suggest that genetic predispositions, in conjunction with environmental triggers, drive the development of both conditions, underscoring the importance of epigenetic research for future therapeutic advancements. 

Pathophysiological mechanisms 

Chronic immunological disorders include both psoriasis and IBD with evidence suggesting high associations between the two [38]. Alterations in intestinal and dermal barrier dysfunction lead to the activation of maladaptive inflammatory immune pathways, which in turn causes dysfunction severe enough to cause IBD and psoriasis [39].

The gut-skin axis refers to the interaction between the gastrointestinal and dermatological systems, mediated by the microbiota of both tissues. Dysbiosis, or the imbalance of microbial communities, is a key factor in both IBD and psoriasis. In IBD, patients often have a reduction in beneficial anaerobes like *Faecalibacterium prausnitzii* and an increase in pathogenic bacteria such as *Proteobacteria*, which drives intestinal inflammation [7]. Similarly, psoriasis patients exhibit dysbiosis in skin microbiota, with an overrepresentation of *Corynebacterium* compared to *Propionibacterium*, often following streptococcal infections that trigger psoriatic lesions [7,40].

The gut-skin axis highlights how dysregulated microbiota can influence systemic inflammation in both the skin and intestines. Microbial metabolites produced by gut bacteria, such as short-chain fatty acids, can modulate immune responses and contribute to the inflammation observed in both conditions [41]. The concept of the gut-skin axis provides a potential therapeutic target for modulating microbiota to treat both IBD and psoriasis, particularly with probiotics, prebiotics, and microbiome-modulating therapies [7,41,42].

**Figure 1:**
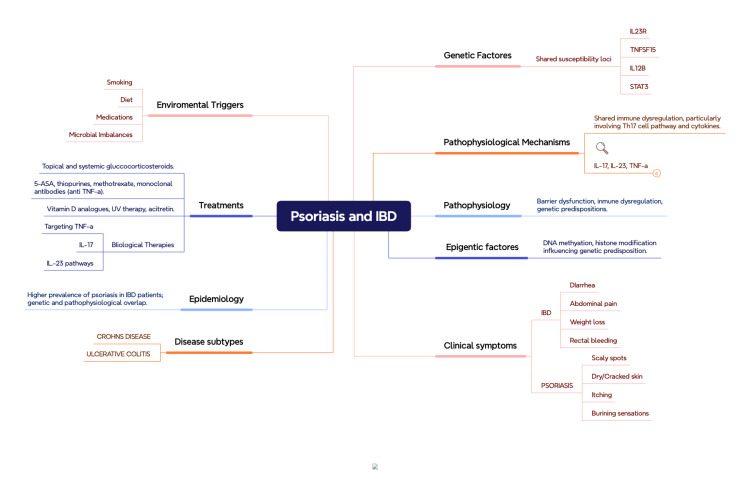
Association between IBD and psoriasis IBD: inflammatory bowel disease

Figure 1 illustrates the interconnectedness of psoriasis and IBD, highlighting shared genetic factors (e.g., IL23R, IL12B), pathophysiological mechanisms (immune dysregulation, cytokines like IL-17, IL-23, and TNF-α), and environmental triggers such as smoking, diet, and microbial imbalances. 

Alterations in the gut microbiome and abnormal immune responses are key factors in the development of IBD, with autophagy malfunction and increased ER stress contributing to CD [39]. Variants in the IL23R gene link the pathophysiology of IBD and psoriasis, where IL-23 plays a critical role in driving immune-inflammatory pathways [43,44]. Genetic and epigenetic modifications, such as CD8+ T-cell DNA methylation and altered MHC and HLA expression, contribute to psoriasis [45]. Additionally, abnormal toll-like receptor expression and dendritic cell activation in both the gut and skin further exacerbate inflammation in IBD and psoriasis, with cytokines like IL-17, IL-22, and IL-12 playing distinct roles in these diseases [43,46].

Clinical implications and shared therapeutic approaches

While there is currently no known curative treatment for IBD and psoriasis, many people can achieve sustained remission with long-term medication. Glucocorticosteroids, which can be given topically, orally, or intravenously, are a staple for IBD flare-ups and can be given topically to treat mild forms of psoriasis. Other treatments include 5-aminosalicylate (5-ASA), antimetabolites, e.g., thiopurines (azathioprine and 6-mercaptopurine) and methotrexate, and monoclonal antibodies, e.g., anti-TNF-α antibodies (e.g., infliximab, adalimumab). Additionally, vitamin D analogues normally used with UV therapy and acitretin, a vitamin A analogue, are effective against psoriasis [47]. Some of the therapeutic strategies include the use of prognostic indicators which uses phenotypic features of the disease to determine its course and prognosis as well as the type of treatment to institute. Prognostic biomarkers are being intensively searched for since phenotype-based disease course prediction has limits in terms of accuracy [48].

The molecular and clinical biomarkers obtained by the medical monitoring and genetic and epigenetic analysis along with clinical data extracted from stratified patient populations become the base of prognostic biomarkers. For instance, In IBD, often by the use of CD8+ T-cell expression profiling, relapse disease has been predicted. This is consistent with "T-cell exhaustion," a condition in which T cells lose their ability to react. A crucial component of individualized treatment in chronic diseases with diverse natural histories will be the use of biomarkers to stratify patients [49,50].

The conventional paradigm for treating both psoriasis and IBD has been a step-by-step process commencing with the least effective treatments, which are additionally the safest; this is known as the "step-up" method, which involves moving up the therapeutic ladder to more potent medicines with associated higher risks [51]. The "top-down" strategy of starting vigorous therapy early in some individuals has been recommended in order to control early inflammation and avoid chronicity. In psoriasis as well as IBD, the afflicted tissue becomes immune-activated due to a confluence of environmental, genetic, and epigenetic variables. Although inflammatory mediators like IL-22 and IL-17 have different functions in IBD and psoriasis as this is also evident in the targeted treatment response, IL-23 and TNF-α seem to play a crucial part in promoting inflammatory response in both disorders [47]. Biologics that target TNF-α are often efficacious in treating both psoriasis and IBD; however, they differ greatly: etanercept, an approved treatment for psoriasis, is ineffective in treating IBD [7]. Moreover, contrary to expectations, the de novo emergence of psoriasis and less commonly IBD has been associated with anti-TNF-α medication. An estimated one case per 1,000 patient-years or 3-10% of patients receiving the treatment have anti-TNF-α-related psoriasis-like symptoms [7]. The most recent production of monoclonal antibodies against IL-17 (brodalumab, ixekizumab, and secukinumab) shows remarkable efficacy in treating moderate to severe psoriasis. In randomized studies, secukinumab and bro-adalimumab were also assessed to treat moderate as well as severe CD patients; however, none of the drugs demonstrated significant efficacy statistically in comparison with placebo. The contradictory findings from IL-17 inhibitory action support the intricate biology of CD in the process of immunity and highlight the significant distinctions in the roles played by Th17 cells in the pathophysiology of IBD and psoriasis [7]. Attention has been drawn recently by the IL-23/Th17 effector axis because of its potential as a therapeutic target and as a key passage to understanding the pathophysiology of psoriasis and IBD due to its synergy with intestinal mucosa cells and keratinocytes.

The broad overlap in treatment protocols between psoriasis and IBD is also rooted in the previously noted immunological commonalities [52,53]. In 2020, Alinaghi et al. carried out a meta-analysis to investigate the correlation along with the frequency of psoriasis and IBD. The prevalence of psoriasis was reported at 3.6% in people having CD while individuals having UC were 2.8% according to the analysis, which included 93 studies. 

On the other hand, 0.5% of UC patients and 0.7% of CD patients had psoriasis [54]. Individuals with psoriasis are more likely to experience IBD, cardiovascular disease, obesity, depression, psoriatic arthritis, diabetes, and many other comorbidities, for instance, depression and immune-mediated illnesses; management of these conditions is difficult for such patients [55]. Even though the pathophysiology and management of psoriasis and IBD are quite similar, each ailment has unique clinical problems. Regarding IBD treatment, there are six biologics that target three distinct molecular targets (IL-23/IL-12, TNF, and anti-integrin therapies): tofacitinib, a tiny chemical, for UC also has the authorization to block the JAK-STAT pathway; however, there are approximately 12 biologic medicines available for the treatment of psoriasis that affect four therapeutic targets (IL-23/IL-12 (p40), TNF, IL-17, and IL-23 (p19)) and an additional modest pharmaceutical inhibitor of phosphodiesterase-4, apremilast. The different treatment landscapes for psoriasis and IBD have had a major influence on clinical practice [47].

Current research and future directions

Gastrointestinal diseases represent a broad spectrum of conditions that present significant diagnostic and therapeutic challenges. Recent research has uncovered shared pathophysiological mechanisms across various gastrointestinal disorders, leading to innovative treatment strategies.

Understanding these common mechanisms is crucial for developing targeted therapies. Studies by Hung et al. [56] highlight the complex relationship between the gut microbiota and the immune system in conditions such as IBD and IBS. Disruptions in the gut microbiota (dysbiosis), impaired mucosal barriers, and immune dysregulation suggest that modulating the gut microbiota holds promise as a therapeutic approach. Similarly, Kumar and Smith [57] emphasize the role of mucosal inflammation and barrier dysfunction in conditions like celiac disease and non-celiac gluten sensitivity, advocating for interventions that restore and protect intestinal integrity.

Recent advancements have identified novel therapeutic targets, offering new avenues for treatment. For instance, Sands et al. [58] discuss the involvement of the JAK-STAT signaling pathway in IBD and propose JAK inhibitors as potential therapies. Chen et al. [59] explore treatments targeting the gut-brain axis in IBS, focusing on serotonin receptors and the communication between the microbiota and the brain. The development of biologic therapies targeting specific cytokines and immune pathways offers hope for precision medicine, tailoring treatments to individual patient profiles to optimize outcomes and reduce risks.

A comprehensive, multidisciplinary approach is essential for managing these complex disorders. Hedin et al. [47] stress the importance of a collaborative care model involving gastroenterologists, dietitians, immunologists, and mental health professionals. This holistic approach ensures that all aspects of a patient's health are addressed. For example, dietitians can offer personalized nutritional plans to support gut health, while mental health professionals help manage the psychological effects of chronic gastrointestinal conditions.

## Conclusions

The correlation between IBD and psoriasis clinically is underscored by shared immunopathogenic mechanisms, involving T-cell dysregulation and pro-inflammatory cytokines like IL-17, IL-23, and TNF-α. Both conditions are influenced by genetic, environmental, and immune factors, with overlapping treatment protocols, including the use of biologics targeting common pathways. Despite unique clinical challenges, understanding these shared mechanisms aids in developing targeted therapies, improving patient outcomes, and managing comorbidities. This intersection emphasizes the crucial need to have an effective multidisciplinary strategy for the management and treatment of these chronic inflammatory illnesses.
